# Delay in diagnosis of tuberculosis in Rawalpindi, Pakistan

**DOI:** 10.1186/1756-0500-4-165

**Published:** 2011-05-26

**Authors:** Muhammad AN Saqib, Irum N Awan, Syed KA Rizvi, Mirza I Shahzad, Zahid S Mirza, Sabira Tahseen, Imran H Khan, Azra Khanum

**Affiliations:** 1 Department of Biochemistry, Pir Mehr Ali Shah Arid Agriculture University Rawalpindi, Murree Road, Rawalpindi-46300, Pakistan; 2 Department of Zoology, Pir Mehr Ali Shah Arid Agriculture University Rawalpindi, Murree Road, Rawalpindi-46300, Pakistan; 3 National Tuberculosis Control Program, Islamabad-44000, Pakistan; 4 Center for Comparative Medicine, University of California, Davis CA-95616, USA

## Abstract

**Background:**

Delay in diagnosis and treatment of tuberculosis (TB) may enhance the chances of morbidity and mortality and play a key role in continuous transmission of the bacilli. The objective of this study was to describe health care seeking behavior of suspected TB patients and initial diagnostic work up prior to consultation and diagnosis at National TB Center (NTC).

**Findings:**

Interviews of 252 sputum smear positive patients were taken from NTC, Rawalpindi. The duration between on-set of symptoms and start of treatment was considered as the total delay and correlated with general characteristics of TB patients. The proportion of males and females were 49.6% and 50.4% with median age of 25 and 24 years respectively. A median delay of 56 days (8 weeks) was observed which was significantly associated with age, cough and fever. More than 50% of the current patients had a history of contact with previously diagnosed TB patients. The majority of patients (63%) visited health care providers within three weeks of appearance of symptoms but only thirty five percent were investigated for TB diagnosis.

**Conclusion:**

Cough and fever are being ignored as likely symptoms of TB by patients as well as health care providers resulting in delay. Engaging private practitioners through public private mix (PPM) approach for expansion of TB diagnosis and increasing public awareness could be more beneficial to reduce delay.

## Background

Tuberculosis (TB) is as ancient as civilization and leading cause of death world wide. Reemergence and association with acquired immunodeficiency syndrome have made TB a global threat. One third of world's population is infected with *Mycobacterium tuberculosis *resulting in 1.8 million deaths annually [1]. TB is more common in developing countries and is associated with unawareness, poor resources and lack of infrastructure for control of disease [2]. Various socio-economical factors such as poverty, migration, over-crowding etc have complicated this deadly disease.

A major issue in TB control and its eradication is delay in diagnosis and commencement of treatment [3]. An undetected and untreated patient has the potential to infect and transmit TB to many individuals daily and results in enhance infectivity [4]. In order to stop TB, it is mandatory to eradicate its source and decrease the chances of transmission from one individual to other. It is, therefore, important to diagnose and treat the infected individual as early as possible.

The magnitude and factors associated with delay were categorically described from many developing as well as developed countries [5]. It was reported that health seeking behavior, low access to health care facilities, poverty, rural residence, old age, alcohol or substance abuse were significant factors associated with delay [6]. Both patients as well health care systems are responsible in this delay process.

Pakistan is currently at 8^th ^place according to TB burden ranking [7]. The low case detection rate observed in the Eastern Mediterranean Region is mainly because of low detection rate in Pakistan and Afghanistan [8]. If this remains unexplored, then a major out break of TB can be expected in Pakistan.

Coughing and fever are two most common symptoms of TB. However coughing is prevalent in general population and is associated with many other illnesses. Both symptoms are not usually considered as causes of TB by patients as well as health care providers which results in delay process. The objective of this study was to describe health care seeking behavior of suspected TB patients and initial diagnostic work up prior to consultation and diagnosis at NTC. This might be helpful in defining and formulating national policy to improve early diagnosis and eventually to reduce TB burden.

## Methods

This study was conducted at NTC, Rawalpindi, Pakistan which is one of the oldest TB centers in the city. Most patients come from Rawalpindi-Islamabad and its adjoining areas while few from other cities of Punjab and Khyber Pakhtunkhwa (KP).

### Data Collection

A total of 252 new sputum smear positive TB patients were enrolled in this study from December 2007 to March 2008 according to a pretested questionnaire (Additional file 1). Information about sociodemographic characteristics, presence and duration of symptoms and history of initial diagnostic work up were collected. To make sure that patients were newly diagnosed, every individual was asked twice for previous TB treatment. The duration of cough was used to find out total delay i.e. time between on-set of cough and final diagnosis as well as start of treatment at NTC. All interviews were made by authors themselves in local language i.e. Urdu. This study was approved by Ethics Committee of Pir Mehr Ali Shah Arid Agriculture University Rawalpindi.

### Statistical Analysis

Data collected was double entered in, cleared and coded using Epi-Info Version 3.5.2 (Center for Disease Control and Prevention, Atlanta, GA., USA) and analyzed with SPSS 16.0. The data was evaluated by using different cut-off points such as 8, 10 and 12 weeks. Since no difference was found, therefore 8 weeks was used as final cut off for the analysis. The group difference was calculated by using Mann-Whitney test and differences were considered statistically significant if P ≤ 0.05. Univariate logistic regression analysis was performed to find out the affect of different demographic variables on total delay. The independent variables were age, sex, fever, family history.

## Results

### General Characteristics of Patients

A total of 252 sputum smear positive patients were interviewed at NTC, Rawalpindi. Out of which 181 (72%) patients were resident of Rawalpindi and Islamabad while rest approached from Rawalpindi Division and other cities of Punjab and KP. The proportion of male and female patients was nearly equal i.e. 49.6% and 50.4% having a median age of 25 and 24 years respectively. The overall age of 79% patients was ≤ 35 years. It was found that females with the age group of 15-19 years were affected more significantly (42.1%) as compared to males (26.7%). Majority of respondents were skilled workers 42% followed by housewives 25%, students 14%, unskilled workers and unemployed 10% and government servants 4%.

### Symptoms and Previous History

The frequency of symptoms in patients recorded was 100% cough, 93% fever and 27% heamoptysis. Bacillus Calmette Guerin (BCG) scar was observed in only 18% patients. Among 252 smears positive patients, 135 (54%) patients had a history of contact with TB patients. About 67% had TB in their immediate family members such as father, mother, sister and/or brother, 26% in close relatives like uncle, aunty and cousin and 4% in friends, neighbors or colleagues.

### Initial Management of Patients

Prior to visiting NTC, only 92 (37%) patients were investigated for diagnosing TB in which 22% had X-ray examination, 10% sputum smear test and 5% with skin test. However patients, who were subjected to these tests, did not get benefited in terms of diagnosis and treatment over those who were not examined. Only 2 patients were examined with all these tests collectively, 15 with X-ray plus sputum test, 5 with X-ray plus skin test and 3 with sputum plus skin test.

### Total Delay

A total median delay of 56 days (8 weeks) was observed with mean delay of 81 days. Patient with age >35 year had higher median delay than those with age 15-35 years. Major difference was observed in different community groups. Unemployed and unskilled workers had 12 weeks median delay as compare to skilled workers and government servant i.e. 8 and 9 weeks respectively. It is noteworthy that students had only 4 weeks median delay (Table 1). However it is important to point out that more than one year delay was also noted in 55 patients (26%). Majority of patients 167 (66%) received some kind of consultation while 43 (17%) did not go to any health care providers before visiting NTC. Only 42 (16%) patients reported directly to NTC and most of them were between 15-34 years. The patients living in Rawalpindi/Islamabad or in surrounding cities visited NTC equally. Univariate logistic regression analysis indicated that age (OR 1.9, 95% CI 1.0-1.3), residence (OR. 1.5 95% CI 0.6-3.4) and sputum test (OR 1.8 95% CI 0.8-4.1) (Table 2) are major predictor for long total delay.

**Table 1:** General Characteristics of Patients

Description	N (%)	Median Delay(Weeks)	P (<0.05)
Total	252	**8**	

**Age (years)			**0.01**

15-35	200 (79)	**8**	

>35	46 (18)	**10**	

**Sex**			**0.04**

Male	123 (49.6)	**6**	

Female	129 (50.4)	**8**	

**BCG**			0.29

Yes	70 (18)	**8**	

No	84 (33)	**8**	

**Cough			**0.00**

Yes	252 (100)	8	

No	0	0	

**Fever			**0.00**

Yes	234 (93)	**8**	

No	18 (7)	**8**	

**Heamoptysis**			

Yes	68 (26)	**8**	

No	184 (73)	**8**	

**Family History**			0.89

Yes	135 (54)	**8**	

No	111 (44)	**8**	

**Relationship with Affected**			0.21

Family	90 (67)	**8**	

Others	38 (28)	**6**	

**Profession**			0.83

Skilled worker	89 (42)	**8**	

Unskilled worker	19 (8)	**12**	

Housewife	52 (25)	**8**	

Students	35 (14)	**4**	

Unemployed	26 (10)	**12**	

Govt. Servants	9 (4)	**9**	

**Residence**			0.36

Rawalpindi/Islamabad	182 (72)	**8**	

Others	50 (20)	**8**	

** Significant p < 0.05

**Table 2:** Relationship between Total Delay and General Characteristics of Patients

Covariate	n	Delayed	Not Delayed	OR (95% CI)
**Age (years)**				

15-35	200 (79%)	69 (35%)	131 (65%)	1.9 (1.0-3.6)
	
>35	46 (18%)	23 (50%)	23 (50%)	

**Sex**				

Male	123 (49%)	54 (44%)	69 (56%)	0.6 (0.3-1.0)
	
Female	129 (51%)	40 (31%)	89 (68%)	

**BCG**				

Yes	70 (46%)	25 (36%)	45 (64%)	0.7 (0.4-1.4)
	
No	84 (55%)	37 (86%)	47 (14%)	

**Fever**				

Yes	234 (93%)	88 (84%)	146 (16%)	0.8 (0.3-2.3)
	
No	18 (17%)	6 (78%)	12 (22%)	

**Heamoptysis**				
Yes	68 (27%)	28 (84%)	40 (16%)	1.0 (0.4-2.5)
	
No	184 (73%)	66 (83%)	118 (17%)	

**Family History**				

Yes	135 (55%)	51 (82%)	84 (18%)	1.0 (0.6-1.7)
	
No	111 (45%)	41 (85%)	70 (15%)	

**Residence**				

Rawalpindi/Islamabad	182 (72%)	70 (83%)	112 (17%)	1.5 (0.6-3.4)
	
Others	25 (20%)	12 (82%)	13 (18%)	

**Skin Test**				

No	237 (95)	89 (38%)	148 (62%)	0.7 (0.2-2.5)
	
Yes	13 (5%)	9 (69%)	4 (31%)	

**Sputum Test**				

No	225 (90%)	81 (36%)	144 (64%)	1.8 (0.8-4.1)
	
Yes	24 (10%)	12 (50%)	12 (50%)	

**X-Ray**				

No	194 (78%)	69 (36%)	125 (64%)	0.7 (0.3-1.8)
	
Yes	55 (22%)	24 (44%)	31 (56%)	

## Discussion

A number of reports have highlighted the presence of delay in diagnosis and start of treatment in TB patients. The factors found to be associated with delay related to patients were health seeking behavior, awareness, poverty and smoking [9]. Similarly diagnosis and treatment of TB was also delayed because of inefficient diagnostic facilities, incompetent health care providers and atypical presentation of patients [10]. In this study, we have focused on the presence of potential TB symptoms especially cough and fever and described the investigation, behavior of patients and health care providers towards these symptoms. We recorded total delay of 56 days which is consistent with reports from neighboring countries of Pakistan such as India (62 days) [11], Bangladesh (60 days) [12], Iran (93 days) [13] and also from other countries like China (71 days) [14], Malaysia (90 days) [15], Japan (78 days) [16], Syria (55 days) located in Asia [17]. An overall average total delay existing in different countries has been calculated as 72 days from 58 reports published in different journals [18]. Similarly, Sreeramareddy *et al*. [19] reviewed 52 different studies and reported 25-155 days average total delay.

TB is commonly associated with coughing, fever and heamoptysis. These symptoms are non-specific and are common in other chest diseases which provide a big challenge for efficacy of any health system [20]. It has been reported that delay in diagnosis of TB is also associated with coexistence of coughing and other lung diseases [21]. Appearance of these symptoms varies and inconsistency in cough and fever was seen. Since the patients gave little importance to seek health care for these symptoms therefore it resulted in delay of diagnosis and treatment. In our study, majority of respondents were residents of Rawalpindi and Islamabad and had easy access to NTC and other public health facilities providing TB care. Although a significant proportion consulted health care providers but only few reported directly to the NTC. A large number of patients had a history of tuberculosis in their families but many of them did not consider TB as the underlying cause of their symptoms. It has been reported that more than 50% of patients received self medication, 42.2% consulted drug stores and only few went to health care providers in Pakistan [8]. It has also been pointed out that the behavior of patients towards TB symptoms was inadequate [22].

Our data further showed that those patients who directly contacted NTC were not only diagnosed but also put on treatment within 3 days. This indicates that patients approaching health facilities equipped for providing free TB care may enhance diagnosis and treatment. However overcrowding at public health centers, the attitude of staff and lack of trust on treatment, discourage patients from visiting these facilities. In general, TB is diagnosed from sputum examination, chest X ray and skin test but unfortunately these are not being used in general practice to exclude TB by health care providers. This shows lack of awareness or interest to follow national guidelines for TB diagnosis. This is consistent with previous study from Pakistan in which only 21% patients were investigated for TB by private doctors [23]. It has also been reported from different countries that mostly doctors did not request investigation. Even those who were investigated, were not diagnosed either because of non specificity of diagnostics methods or incompetence of health care providers. There is need for engaging private practitioners through public private mix (PPM) approaches for expansion of TB care. This will improve diagnosis and treatment of patients approaching private practitioners for these symptoms.

Previous exposure of TB is an important source of infection which is most common in Pakistan. Majority of patients had strong contact history still most of them were not suspected for TB. Health care providers either did not enquire about TB contact history or respond appropriately indicating casual attitude. Furthermore contact based investigations for TB are needed to enhance diagnosis.

The magnitude of total delay has been described which was too high and documented as 90 days in Multan and Sialkot [24] and 97 days in Karachi [8]. It has been observed that percentage of male and female, age, frequency of cough and fever and previous exposure to TB patients are nearly similar in these major cities. Similarly prevalence of TB in different occupational groups of society was also same as majority of them were skilled workers (21-45%), housewives (25-40%) and students (9-15%). This similarity provides an opportunity to launch a massive campaign at national level to overcome and to formulate intervention leading to early diagnosis.

According to WHO, TB is more common in males than females with a ratio of 2:1 [1]. In a recent report, it has been shown that this sex biased observation in TB might be due to the biological differences between both sexes [25]. But our data revealed that the proportion of males and females patients is nearly equal. This was supported by a study carried out in Iran which mentioned that pattern of disease in high epidemic region is not consistent and variations may exist [26].

This study has some limitations. Firstly patients were asked for the presence and duration of potential TB symptoms i.e. cough, fever and heamoptysis rather than TB itself. Secondly all data is based on patients' memory therefore they might have either missed or mislead a question. Similarly usage of regression analysis with skewed data might affect interpretation of results. Of importance, study was conducted in NTC and thus not representing those patients who either visited other TB care centers or private doctors only.

## Conclusions

Our study showed that most common symptoms of TB like coughing and fever are often ignored by patients as well as health care providers resulting in delay. A significant association of total delay with coughing and fever were observed providing an opportunity to investigate patients for TB having these symptoms for more than three weeks.

Although NTP has improved the number of patients by implementation of DOTS strategy in public sectors health facilities but this study showed high magnitude of total delay in diagnosis and start of treatment in TB patients. Therefore an active engagement of private practitioners and other health care providers through PPM approaches is necessary. This will give equity of access to TB patients seeking care for TB symptoms.

## Competing interests

The authors declare that they have no competing interests.

## Authors' contributions

MANS interviewed the patients, organized and interpreted the data and wrote the manuscript independently. INA, SKA and MIS also interviewed the patients. INA, MIS, ST, IHK and AK collectively designed the questionnaire. ZSM along with MANS performed the statistical analysis. AK conceived the study, helped in manuscript drafting and revised final draft critically. ST also helped during write up of manuscript. All authors have approved manuscript.

## Supplementary Material

Additional file 1 **The questionnaire**. This contains all questions which we asked patients during interviews. Click here for file
