# Supplementary material for: Delay in diagnosis of tuberculosis in Rawalpindi, Pakistan
Source: BMC Res Notes. 2011 May 26;4:165. doi: 10.1186/1756-0500-4-165 (PMC3123219; doi:10.1186/1756-0500-4-165)
Supplement: Additional file 1 — The questionnaire. This contains all questions which we asked patients during interviews. [file 1756-0500-4-165-S1.DOC]

**CLINICAL INFORMATION FORM**

Code:

**A IDENTIFICATION OF PATIENT**

Name: ______________________________

Registration No: __________________

TB district number __________________

Date registered __________________ ____ ____ ____ Day Month Year

Sputum smear test Result: _____________

Sex: Male ________ Female ________

Age: _______________

BCG SCAR: YES ________ NO ________

Symptoms Duration:

- Cough Yes ________ No ________ _______
- Fever Yes ________ No ________ _______
- Haemoptysis Yes ________ No ________ _______

History of TB in last: Yes________ No__________

1. Monthly income / Profession _________________________________________

2. Clinical History: ________________________________________________

3. Routine investigation: ________________________________________________

**B HISTORY GIVEN BY THE PATIENT**

**B1** Previously treated for TB? Yes ________ No ________

**B2** **Standardized history**

- Skin test result (tuberculin skin test)? ____________________________________
- Did you have X-ray examination prior to this episode? __________________
- Did you have sputum examinations prior to this episode? __________________
- Did you ever take tuberculosis drugs for more than one month? If yes what was name? __________________________________________________________________

__________________________________________________________________

- Did you have injections for more than one month? ________________________
- TB in family, if yes how many members? ________________________________
- Did you have some consultation? ______________________________________
- Did the patient remember previous treatment for TB after these questions?

No ________ Yes ________
